# COVID-19 rise in Bangladesh correlates with increasing detection of B.1.351 variant

**DOI:** 10.1136/bmjgh-2021-006012

**Published:** 2021-05-05

**Authors:** Senjuti Saha, Arif M Tanmoy, Yogesh Hooda, Afroza Akter Tanni, Sharmistha Goswami, Syed Muktadir Al Sium, Mohammad Saiful Islam Sajib, Roly Malaker, Shuborno Islam, Hafizur Rahman, Ataul Mustufa Anik, Nikkon Sarker, Mohammad Shahidul Islam, Kinkar Ghosh, Probir Kumar Sarkar, Mohammed Rizwanul Ahsan Bipul, Syed Shafi Ahmed, Mohammod Shahidullah, Samir K Saha

**Affiliations:** 1 Child Health Research Foundation, Dhaka, Bangladesh; 2 Cell Biology, MRC-Laboratory of Molecular Biology, Cambridge, UK; 3 Dhaka Shishu Hospital, Dhaka, Bangladesh; 4 Department of Pediatrics, Bangladesh Institute of Child Health, Dhaka, Bangladesh; 5 Department of Neonatology, Bangabandhu Sheikh Mujib Medical University, Dhaka, Bangladesh; 6 Department of Microbiology, Bangladesh Institute of Child Health, Dhaka Shishu Hospital, Dhaka, Bangladesh

**Keywords:** COVID-19, SARS, vaccines

Epidemiological, phenotypic and genomic characterisation of certain variants of SARS-CoV-2 have highlighted the changing transmissibility, infectivity and antigenic escape capability of this virus. Of considerable interest are the B.1.1.7 variant (20I/501Y.V1)^1^ and B.1.351 variant (20H/501Y.V2)^2^ that have now been reported from multiple countries around the world. B.1.1.7 was first detected in September 2020 in the UK through genomic surveillance, and it contains a mutation (N501Y) in the receptor-binding domain of the spike protein that has been reported to increase transmission^1^ and virulence^3^ through genomic and epidemiological studies. The variant still shows strong response to antibody treatment and is effectively neutralised by antibodies generated on vaccination by mRNA-based vaccines.^4^ The B.1.351, first identified in South Africa in October 2020,^2^ carries the N501Y mutation and two additional mutations (E484K and K471N) that confer increased antibody resistance.^4^ These findings make it imperative to continuously track circulating variants of SARS-CoV-2 globally, especially in low-resource settings, to institute evidence-based policy decisions. This editorial aims to raise awareness regarding the increasing isolation of the B.1.1.7 and B.1.351 variants and documented cases of re-infection in Bangladesh in recent months.

Bangladesh, one of the most densely populated countries of the world, detected the first SARS-CoV-2 cases on 8 March 2020. Immediately following that, non-pharmaceutical interventions including school closures, work from home mandate and flight bans were instituted for the following 2 months (figure 1A). After the first peak in July 2020 (3810 cases/day, 7-day average) case numbers reduced, with a slight surge in December 2020 (2185 cases/day, 7-day average) that also subsided.^5^ A small study conducted in July of 2020 estimated that seroprevalence in Dhaka, the capital of Bangladesh, ranged from 45% to 74%.^6^ However, starting in early March 2021, case numbers began to rise rapidly, and the reproduction number is estimated to have doubled from 0.79 on 21 January 2021 to 1.66 on 23 March 2021 (figure 1B). The average test positivity rate in the last week of March 2021 was 17% country-wide, when the average number of tests conducted was 26 127/day.^7^ Bangladesh introduced the ChAdOx1 (Oxford-AstraZeneca) vaccine on 27 January in 2021, and as of 13 April 2021, 5.6 million people (of the 164 million population) have received the first dose of the vaccine, and 733 000 have received the second dose.^7^

**Figure 1:**
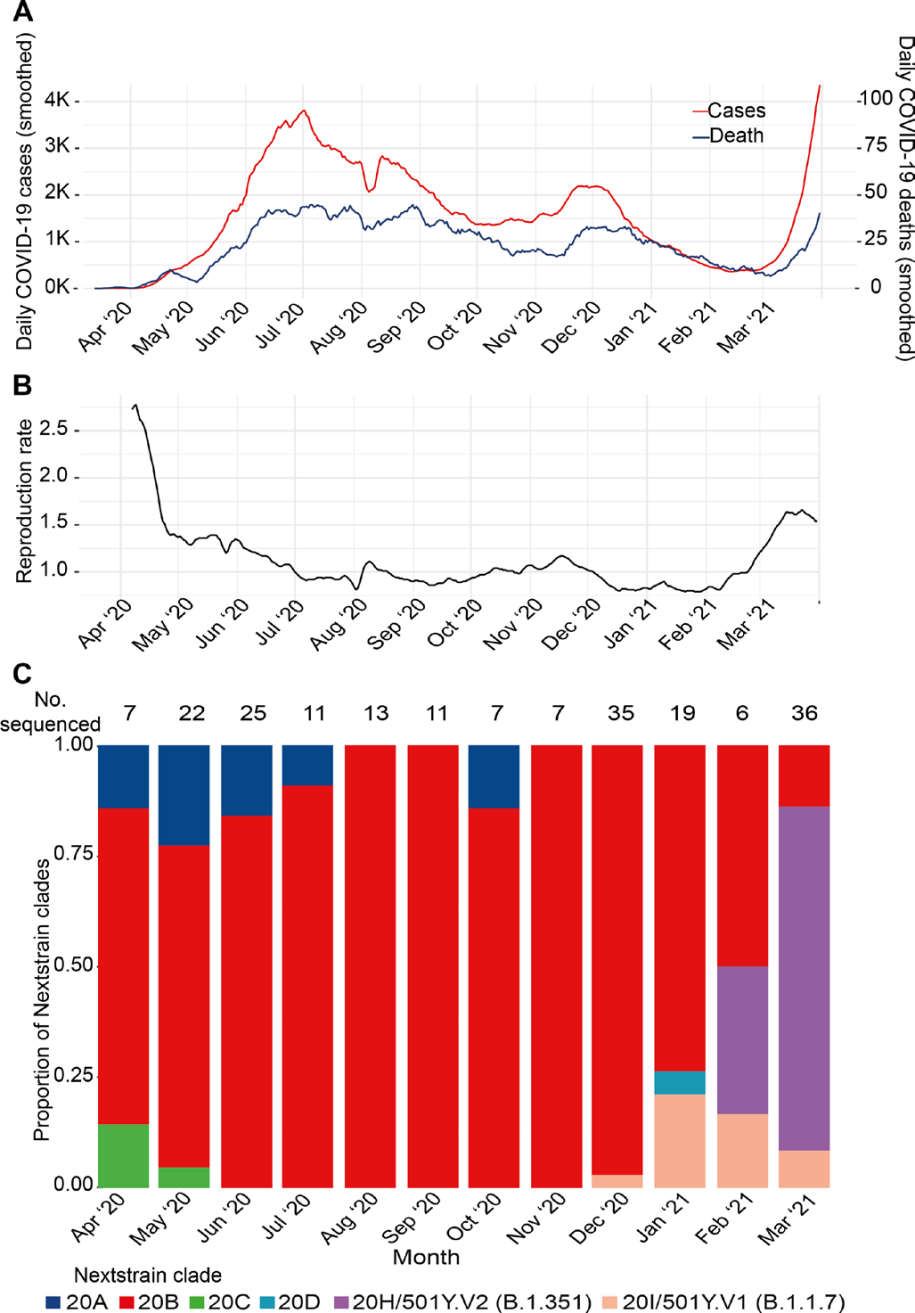
COVID-19 cases in Bangladesh and trends in circulating lineages of SARS-CoV-2. (A) The 7-day average of confirmed COVID-19 cases and deaths. (B) The reproductive rate (R_0_). Data for (A) and (B) were obtained from Our World in Data (https://ourworldindata.org/coronavirus). (C) The Nextstrain clades (Pangolin lineage) of the 204 SARS-CoV-2 sequenced at the Child Health Research Foundation.

Our group at the Child Health Research Foundation (CHRF) functions as a COVID-19 testing and sequencing centre based in Dhaka, the capital of Bangladesh.^8^ From April 2020 through 30 March 2021, we have identified >12 000 cases in our testing facility, of which we have sequenced 204 SARS-CoV-2 genomes. All PCR tests are conducted using the TaqPath COVID-19 Combo Kit (Thermo Fisher Scientific, A47814, Waltham, Massachusetts, USA), SARS-CoV-2 sequencing libraries are prepared using the ARTIC V.3 protocol, paired-end reads are generated using iSeq 100, and finally, consensus genomes are assembled using the IDSeq platform of the Chan Zuckerberg Initiative, as described elsewhere.^9^ Samples are usually randomly selected from positive tests; in addition, we may sequence when case history suggests possible antigenic escape. Genomes with metadata are uploaded on GISAID.^10^ Additionally, we have developed and regularly maintain a Bangladesh-specific build in Nextstrain^11^ of all SARS-CoV-2 isolates sequenced and uploaded to GISAID from entire Bangladesh.^12^

The monthly distribution of the Nextstrain clades of SARS-CoV-2 sequences from our testing facility are shown in figure 1C. Sequenced samples were randomly selected from positive cases with cycle threshold value <30, except six cases of possible re-infection or antigenic escape throughout the surveillance. The sequenced samples represented all eight administrative divisions of Bangladesh. We first detected the B.1.1.7 variant in December 2020 in our genomic surveillance. In the following 3 months of January, February and March 2021, 21% (4/19), 16% (1/6) and 8% (3/36) of the genomes sequenced belonged to the B.1.1.7 lineage in our genomic surveillance (9 total). We identified B.1.351 variant in February 2021; in February and March, 33% (2/6) and 77% (28/36) of the genomes sequenced belonged to the B.1.351 variant (30 total). The sequenced genomes are a small proportion of confirmed SARS-CoV-2 cases in the country, and hence may not reflect the diversity of SARS-CoV-2 circulating in Bangladesh. However, such genomic data collected over the entire period of the pandemic are overall scarce from South Asian countries; this surveillance provides us clues as to why cases may be rising in the entire region. In addition, new genomes being uploaded to GISAID by other groups in Bangladesh indicate a similar rise in variants. An additional 100 sequences were uploaded on GISAID by two independent groups in March 2021, where 72 belonged to B.1.351 (72%) and 10 to B.1.1.7 (10%).

## Two cases of second infection by B.1.351 and two cases of infection after vaccination using ChAdOx1

All healthcare workers of CHRF are regularly screened for SARS-CoV-2 infection using RT-PCR. In March 2021, 10 healthcare workers tested positive; samples from 4 cases were sequenced as possible cases of antigenic escape, all of which were B.1.351, and whose details are provided below.

### Cases of reinfection by B.1.351

Of the four cases of B.1.351 detected among CHRF healthcare workers, two were cases of reinfections. Throughout our genomic surveillance, we sequenced a total of three possible cases (six samples) of reinfection, one in July 2020, and two in March 2021; the latter two are described here. Case 1 (32 years old, female) was first confirmed to have an infection on 21 July 2020, with two consecutive positive tests over 7 days. Following resolution of symptoms, there were 12 negative tests over 7 months. On 21 March 2021 (routine screening), case 1 tested positive again. Case 2 (28 years old, male) was first confirmed to have an infection on 23 June 2020, with three consecutive positive tests over 17 days. Following resolution of symptoms, there were 10 negative tests over 8 months. On 21 March 2021 (routine screening), case 2 tested positive again. Using genome sequencing, it was confirmed that in both cases, the first episode was caused by the Nextstrain clade 20A, and the second episode was by 20H/501Y.V2 (B.1.351). Neither of the cases received a vaccine. In both cases, the first and the second episodes were mild and did not require hospitalisation or any supplementary oxygen support.

### Cases of infection after first dose of ChAdOx1

During this surveillance, two cases of infection by B.1.351 were noted within the healthcare workers who received the first dose of ChAdOx1. We sequenced samples from both cases. Case 3 (24 years old, male) and case 4 (27 years old, female) did not have any history of previous infection (either symptomatic or asymptomatic detected through regular screening) and received the first dose of ChAdOx1 on 14 and 16 February 2021, respectively. They both tested positive for SARS-CoV-2 B.1.351 during routine screening on 22 March 2021, 36 and 34 days after vaccination. Both cases were asymptomatic.

## Concurrent increase of variant detection and cases have important implications for vaccine policies

The isolation of these variants correlates to the rapid increase in the number of SARS-CoV-2 cases and test positivity rate in Bangladesh. Genomic surveillance must continue to monitor this trend as these findings have grave implications for mitigation and vaccination policies and must be considered by policy makers when designing public health interventions. We recommend re-introduction of strict non-pharmaceutical interventions to prevent further spread of these variants within Bangladesh and rise of new ones.

Recent evidence about the ChAdOx1 vaccine suggests that it provides limited protection against mild-moderate COVID-19 caused by the B.1.351 variant.^13^ However, concrete data on its efficacy in reducing severe disease is pending. This has led to questions about the circulating variants in Bangladesh, decisions on ongoing and future vaccine policies, and whether ChAdOx1 can help mitigate the burden on hospital beds. Considering the resource-constrained health system of Bangladesh, any vaccine that provides protection, at least against the severe COVID-19 cases, will decrease the burden on the limited hospital and intensive care unit beds in the country. At the same time, introduction of updated or different vaccines may need to be considered as more data become available. This is particularly important for low-income and middle-income countries like Bangladesh that, unlike high-income countries, have had limited options for vaccine access to date. Data on efficacy of ChAdOx1 on severe COVID-19 caused by the B.1.351 variant is urgently needed.

### Patient public involvement

Research questions related to SARS-CoV-2 detection and sequencing were designed according to the public health needs of Bangladesh. COVID-19 test results are immediately confidentially shared with the Directorate General Health Services, Government of Bangladesh, who share them with the patients. Anonymised sequencing results are uploaded on online open-access platforms, GISAID and Nextstrain. Results of samples collected from CHRF healthcare works are shared with all participants in real time in Bangla and English.

## Data Availability

Data are available in a public, open access repository.
